# Controls on Erosion and Cyclic Step‐Formation Upstream of Waterfalls

**DOI:** 10.1029/2024GL110751

**Published:** 2024-11-22

**Authors:** T. Inoue, Y. Hiramatsu, J. S. Scheingross, S. Yamaguchi, K. Takahashi

**Affiliations:** ^1^ Graduate School of Advanced Science and Engineering Hiroshima University Hiroshima Japan; ^2^ Civil Engineering Research Institute for Cold Region Hokkaido Japan; ^3^ Department of Geological Sciences and Engineering Nevada Geosciences University of Nevada Reno NV USA; ^4^ Faculty of Engineering Hokkaido University Hokkaido Japan

**Keywords:** waterfall, cyclic steps, bedrock, erosion

## Abstract

Waterfall retreat transmits base‐level perturbations upstream, thereby providing markers of changing climate and tectonics. In homogeneous rock, waterfalls often retreat either by direct waterfall‐face erosion or incision from repeating (‘cyclic’) steps formed above waterfalls. We lack knowledge on the conditions driving these different erosion styles, limiting our ability to predict waterfall retreat. We address this knowledge gap through flume experiments assessing how changing flow hydraulics modulates bedrock erosion. We show that, under large discharges, changes in flow hydraulics cause spatial variability in particle impact velocity, leading to cyclic step formation. As discharge decreases, both the magnitude and spatial variability of particle impact velocity decreases, causing more uniform erosion, limiting cyclic step development and potentially allowing direct erosion of the waterfall face to become the dominant retreat mechanism. These results suggest climate change and water‐resource management can alter the rate and style of waterfall retreat.

## Key Points

- The spatial distribution of particle impact velocity above waterfalls is sensitive to flow hydraulics
- At large discharges, increased spatial variability in particle impact velocity drives cyclic step formation above waterfalls
- At low discharges, reduced variability in impact velocity limits cyclic step formation and promotes retreat via waterfall‐face erosion

## 1 Introduction

Changes in river base‐level can form steepened river profiles (termed knickpoints), which often include waterfalls (e.g., Cook et al., 2013; Crosby & Whipple, 2006). Waterfalls can drive upstream knickpoint retreat (e.g., Berlin & Anderson, 2007; Howard et al., 1994; Mackey et al., 2014), while lowering riverbed elevation, triggering hillslope steepening and, in cases, landsliding (Baynes, Lague, & Kermarrec, 2018; Gallen et al., 2011; Golly et al., 2017). Thus, waterfall retreat offers an approximation of landscape response rate to base‐level change (e.g., Crosby & Whipple, 2006; Howard et al., 1994; Whittaker, 2012), and understanding waterfall retreat mechanisms and rates allows improved predictions of landscape evolution following base‐level perturbations.

Waterfalls typically retreat via direct waterfall‐face erosion (e.g., undercutting (Gilbert, 1890; Haviv et al., 2010)), toppling and plucking of jointed‐rock blocks (e.g., Anton et al., 2015; Dubinski & Wohl, 2013; Lamb & Dietrich, 2009), or vertical incision of repeating steps and pools, termed cyclic steps, which form where flow accelerates toward waterfall lips (e.g., Baynes, Lague, Attal, et al., 2018, Baynes, Lague, & Kermarrec, 2018; Inoue et al., 2023; Lamb et al., 2007; Scheingross et al., 2019).

Here, we focus on cyclic‐step retreat and direct waterfall‐face erosion, specifically investigating when each mechanism dominates in homogeneous rock. Previous experiments show cyclic steps can form and develop into waterfalls under constant sediment and water flux (Scheingross et al., 2019), and cyclic‐step‐induced waterfall retreat may cause knickpoint self‐formation in the absence of base‐level perturbations (Rothman et al., 2023). Cyclic steps preferentially form above waterfalls with modest drop heights ($H_{drop}/h$ ≈ 3–6, where *H*_*drop*_ is waterfall height and *h* is flow depth (Inoue et al., 2023; Scheingross et al., 2019)), and this can result in short waterfalls retreating faster than tall waterfalls, the latter of which tend to retreat via headwall undercutting (Inoue et al., 2023).

Waterfall retreat rate via cyclic‐step incision is set by step spacing and vertical‐incision rate (Inoue et al., 2023; Lamb et al., 2007). However, the mechanism forming new steps upstream of the waterfall lip, thereby driving retreat, remains unresolved. Theory (Izumi et al., 2017) suggests that cyclic steps are formed by instabilities in, and feedback between, water flow and sediment transport under Froude supercritical flow, and that cyclic step wavelength is defined by the interval between hydraulic jumps. Existing theory (Izumi et al., 2017) models cyclic step formation on flat beds, but cannot address areas upstream of waterfalls with accelerating flow, steep slopes and high erosion rates (Haviv et al., 2006).

We performed flume experiments investigating cyclic step formation and waterfall retreat. We present results from an erodible‐bed experiment showing waterfall retreat via cyclic‐step development and complementary fixed‐bed experiments measuring flow velocity and sediment transport trajectories under varying flow depth. Combined, these experiments allow evaluation of how flow acceleration above waterfalls modulates sediment impacts and cyclic step formation.

## 2 Methods

**Figure 1:**
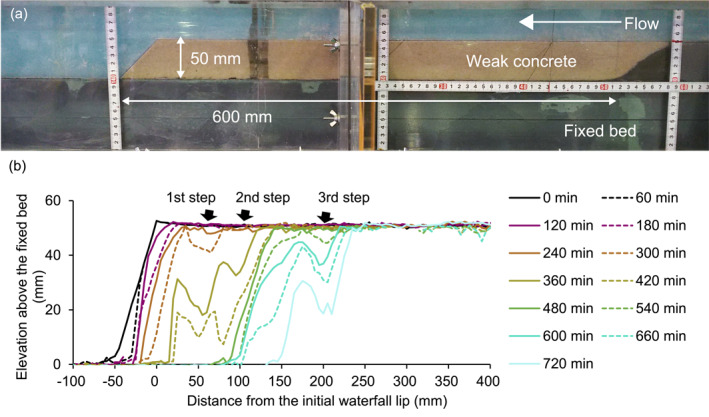
Run A1 (a) side view and (b) bedrock‐bed longitudinal profiles.

We performed two experimental sets. The first set is a paired erodible‐bed (Run A1) and fixed‐bed (non‐erodible) experiment (Run B1) with identical geometry, water discharge and sediment supply (Table S1 in Supporting Information S1). We compared the evolving bedrock morphology and erosion rate in the erodible‐bed experiment to the flow hydraulics and particle impact velocity measurements in the fixed‐bed experiment. We complement the first experimental set with two fixed‐bed experiments (Runs B2 and B3) with identical geometry to the first set, but varying flow depth (Table S1 in Supporting Information S1). All experimental geometries matched the Inoue et al. (2023) Run 1 experiment, except here we narrowed channel width to 0.01 m to allow observations through acrylic sidewalls. We used a 3 m long, rectangular channel with a 45° sloping waterfall installed two m upstream of the outlet (Figure 1a). Following Inoue et al. (2023), we scaled the experiments to ∼1/100th of the Oiran‐buchi waterfall (Sapporo, Japan, 42.9872018, 141.3369729) with a 5 cm drop height (Hdrop), 1.4 mm sediment size (D), and 0.02 channel slope (S).

### 2.1 Paired Erodible‐ and Fixed‐Bed Experiments

#### 2.1.1 Erodible‐Bed Experiment (Run A1)

For the erodible‐bed experiment, we set h = 8 mm, matching Inoue et al. (2023) Run 1 (which produced cyclic‐step driven waterfall retreat). The imposed flow conditions result in $H_{drop}/h$ ≈ 6, within the range associated with cyclic‐step development (Inoue et al., 2023; Scheingross et al., 2019), a normal Froude number (i.e., a Froude number under steady and uniform flow), measured far upstream of the waterfall to avoid the drawdown influence (Haviv et al., 2006), of ${Fr}_{n}$ ∼1.7, and fully turbulent flow (Reynolds number of ∼3,800 and Reynolds particle number of ∼55) (Table S1 in Supporting Information S1).

We used a homogeneous concrete mixture (1:50:8 White Portland cement to 0.34 mm diameter silica sand to water (Inoue et al., 2023; Mishra et al., 2018)) which eroded via particle impacts and did not erode under clear water. We installed a non‐erodible bed below the waterfall, allowing observations of waterfall retreat and cyclic‐step formation without the complication of plunge‐pool undercutting (Figure 1a). In Run A1, we held flow discharge ($Q_{w}$ = 0.038 l/s) and sediment supply ($Q_{s}$ = 6.7 ml/min) constant for the 12 hr experimental duration, measuring bedrock‐bed longitudinal profiles (with deposited sediment removed) every hour using a laser point gauge (∼0.1 mm vertical accuracy, 5 mm spatial resolution).

We designed Run A1 to minimize sediment deposition. The hydraulic roughness height of the initial bedrock surface, $k_{s}$, calculated backward from Manning's formula ($n=1/U\cdot h^{2/3}\cdot S^{1/2}$) and $k_{s}={\left(7.66n\sqrt{g}\right)}^{6}$, was ∼0.54 mm (smaller than the alluvial roughness height of 1.4–5.6 mm (1–4 times particle size)). Here, n is Manning's roughness coefficient, U is depth‐averaged velocity ($U=Q_{w}/hB$), B is channel width, and g is gravitational acceleration. Following Inoue et al. (2014) and Mishra et al. (2020), if bedrock roughness is less than alluvial roughness, sediment will not be deposited unless sediment supply ($Q_{s}$) exceeds sediment transport capacity ($Q_{sc}$). For Run A1, $Q_{s}/Q_{sc}$ = 0.33 in the uniform flow section far upstream from the waterfall when calculating $Q_{sc}$ following Meyer‐Peter and Müller (1948), with Shields number $\tau_{*}=RS/R_{g}D$ where R is hydraulic radius $R_{g}$ is specific gravity of submerged sediment (1.65) and the critical Shields number for smooth bedrock is $\tau_{*c}=0.03{\left(k_{s}/D\right)}^{0.6}$ (Mishra et al., 2020).

#### 2.1.2 Fixed‐Bed Experiment (Run B1)

We paired Run A1 with a fixed‐bed experiment (Run B1) with identical initial geometry and flow conditions (*Q*_*w*_ = 0.038 l/s, h = 8.0 mm, $\tau_{*}/\tau_{*c}$ = 4.1, Table S1 in Supporting Information S1). In Run B1 we measured the flow velocity field using Particle Image Velocimetry (PIV), we measured the rate of particle impacts and water surface slope with high‐speed camera images, and we measured particle impact velocities with Particle Tracking Velocimetry (PTV) (Text S1 and S2). We performed these measurements on the fixed bed, because bed evolution in the erodible experiment was faster than the time needed to capture and save images for PIV and PTV analyses (shooting takes minutes, but it takes hours to save the captured images, thereby drastically limiting the rate at which experiments can proceed). The PIV and PTV analyses allow assessing how changing flow hydraulics and sediment transport modulate the spatial distribution of erosion upstream of waterfalls, and, in turn, may influence whether waterfalls retreat via cyclic‐step incision or erosion of the waterfall face.

### 2.2 Additional Fixed‐Bed Experiment Varying Flow Depth

We compare Run B1 with additional fixed‐bed experiments (Runs B2 and B3), varying flow depth (by varying water discharge), while holding geometry and sediment supply constant. In Run B2 we set h = 6.5 mm ($Q_{w}$ = 0.027 l/s, ${Fr}_{n}$ = 1.64, $\tau_{*}/\tau_{*c}$ = 3.3, $Q_{s}/Q_{sc}$ = 0.51), and in Run B3 we set *h* = 5.0 mm (*Q*_*w*_ = 0.0175 l/s, ${Fr}_{n}$ = 1.57, $\tau_{*}/\tau_{*c}$ = 2.6, $Q_{s}/Q_{sc}$ = 0.93) (Table S1 in Supporting Information S1). The change in flow depth from Run B1 to B3 results in a 1.6 fold difference in excess shear stress, and the 5 mm flow depth in Run B3 is the thinnest reasonable flow depth to maintain saltation of the 1.4 mm diameter sediment. In Runs B2 and B3, we measured particle impact velocity with PTV, but we did not make PIV or water surface slope measurements (Text S1 and S2 in Supporting Information S1).

Runs B1 ($H_{drop}/h$ ≈ 6) and B3 ($H_{drop}/h$ ≈ 10) approximately match Inoue et al. (2023) Run 1 ($H_{drop}/h$ ≈ 6) and Run 2 ($H_{drop}/h$ ≈ 10), which produced waterfall retreat via cyclic‐step incision (Run 1) and via a mix of direct waterfall‐face erosion and cyclic‐step incision (Run 2). However, the Inoue et al. (2023) experiments varied $H_{drop}/h$ by changing waterfall drop height while holding discharge constant, unlike the experiments here (which use constant *H*_*drop*_ and variable *h*). Our experimental set up did not allow replication of Inoue et al. (2023) Run 3 ($H_{drop}/h$ = 25, retreat exclusively via direct waterfall‐face erosion), because large $H_{drop}/h$ ratios required thin flow depths, limiting sediment saltation and transport.

### 2.3 Estimating Erosion From Particle Impacts

We use particle trajectories in the fixed‐bed experiments to calculate particle impact rate and velocity, both of which drive bedrock erosion. Following Sklar and Dietrich (2004), we express bedrock erosion rate E, as,

(1) $$E=V_{i}I_{r}F_{e}$$

where $V_{i}$ is the average volume of rock detached per particle impact, $I_{r}$ is the particle impact rate per unit area and time, and $F_{e}$ is the fraction of bedrock exposed on the bed. Sklar and Dietrich (2004) calculate $V_{i}$ as

(2) $$V_{i}=\frac{1}{2}\frac{mw_{i}^{2}}{\varepsilon_{v}}$$

where m is particle mass, $w_{i}$ is the vertical particle impact velocity and $\varepsilon_{v}$ is a constant reflecting the energy required to erode a unit volume of rock (Sklar & Dietrich, 2001, 2004).

The model of Bitter (1963), on which Sklar and Dietrich (2004) based Eqation 2, sets a minimum, threshold energy for bedrock erosion to occur. Equation 2 can be re‐written including an erosion threshold as,

(3) $$V_{i}=\frac{1}{2}\frac{m\left(w_{i}^{2}-w_{ic}^{2}\right)}{\varepsilon_{v}}$$

where $w_{ic}$ is the threshold impact velocity for erosion.

Spatially variable erosion in our experiments should be driven exclusively by changes in particle impact velocity and rate, because our experiments have zero sediment cover ($F_{e}=1$), hold m constant by using single‐size sediment, and use a single lithology (concrete) which keeps ε_v_ constant. We propose that cyclic steps are most likely to form in areas maximizing either the product of particle impact velocity and impact rate (i.e., $\max\left(w_{i}^{2}I_{r}\right)$) or the excess impact velocity and impact frequency (i.e., $\max\left[\left(w_{i}^{2}-w_{ic}^{2}\right)I_{r}\right]$).

## 3 Cyclic Step Formation Under High Flow

### 3.1 Erodible‐Bed Experiment (Run A1)

We begin by examining the paired Runs A1 and B1. In the erodible‐bed experiment (Run A1), the waterfall retreated primarily via the formation of upstream, vertically drilling cyclic steps (Figure 1b), with a minor role of direct erosion of the waterfall face. Over the first 240 min of the experiment, the waterfall face retreated ∼30 mm with no step development. Between 240 and 300 min, a step formed ∼60 mm upstream of the initial waterfall lip, and then rapidly drilled downwards from 300 to 360 min while a second upstream step began forming and vertically incising (Figure 1b, Table S2 in Supporting Information S1). Vertical step drilling continued until ∼480 min, after which a planar waterfall face transiently formed ∼140 mm upstream of the initial waterfall. By 600 min, the upstream edge of the planar waterfall face was beveled off and a new step formed that continued to vertically drill and promote upstream retreat until the experiment ended at 720 min (Figure 1b). Cyclic steps formed during waterfall retreat at ∼60, ∼100, and ∼200 mm upstream of the initial lip, yielding an ∼67 mm average step spacing (three steps over a 200 mm reach).

The pattern and style of erosion in Run A1 matches Inoue et al. (2023) Run 1, which used a wider flume (with a width to depth ratio of ∼6), similar to natural bedrock channels (Buckley et al., 2024), to allow waterfall retreat with a self‐formed bedrock channel. In both Run A1 and Inoue et al. (2023) Run 1, the first step formed ∼50–100 mm upstream of the waterfall lip, and waterfall retreat was driven by cyclic step formation. Inoue et al. (2023) reports an average step spacing of ∼130 mm for Run 1; however, this includes additional length of the tortuous inner channel that formed during waterfall retreat in their three‐dimensional experiment. Calculating step spacing using a straight‐line retreat distance for Inoue et al. (2023) Run 1 yields an ∼80 mm average step spacing (6 steps formed over ∼480 mm of straight‐line retreat), similar to the ∼67 mm step spacing in Run A1. The consistency between Inoue et al. (2023) Run 1 and Run A1 suggests that narrowing the flume did not alter the rate and style of bedrock erosion observed here, despite the fact that Run A1 had width to depth ratios significantly smaller than in natural channels.

### 3.2 Fixed‐Bed Experiment (Run B1)

**Figure 2:**
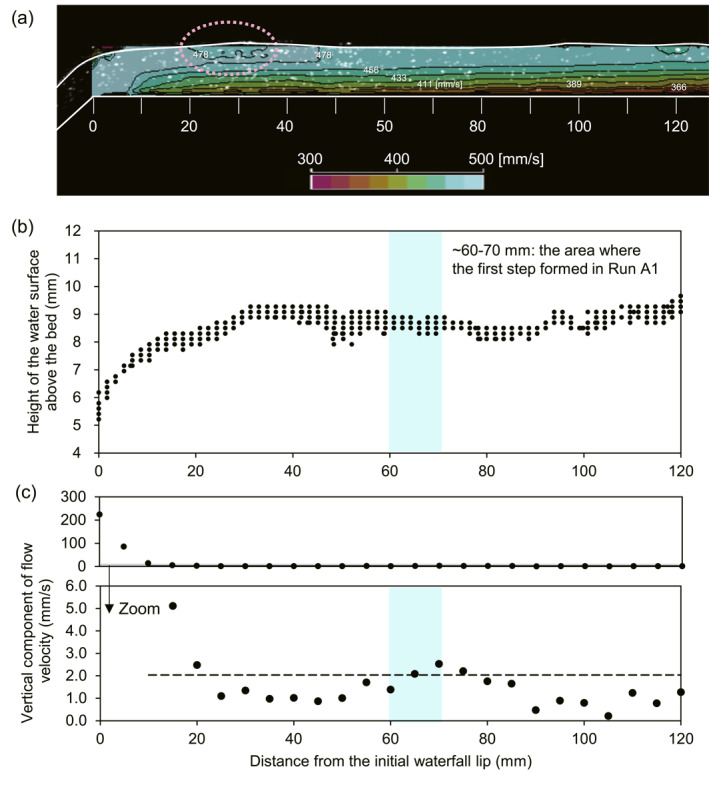
Run B1 flow hydraulics. (a) Horizontal component of time‐averaged flow velocity, white lines represent the water and fixed‐bed surface, white dots are resin particles. The pink circle shows a local reduction in near‐surface flow velocity relative to deeper flow. (b) Water surface profile (measured from 20 images showing temporal variability). (c) Vertical component of flow velocity. Top plot shows all data and bottom plot shows zoom in; the dashed line is the mean vertical component of flow velocity (2.0 mm/s) measured from 10 to 120 mm.

Run B1 allows examining how flow hydraulics, particle impact rate and impact velocity influence cyclic step development under conditions identical to the start of Run A1. PIV analyses show both spatial acceleration and thinning of water flow toward the waterfall lip (Figure 2a), consistent with the expected draw down approaching a free overfall (Rouse, 1936, 1937). However, the flow acceleration and thinning were not monotonic. Whereas, at most locations upstream of the waterfall horizontal flow velocity increased monotonically with distance above the bed, at ∼25–35 mm upstream of the waterfall, there was a velocity reversal, with flow at the surface at 456 ∼ 478 mm/s, slower than flow immediately below it (>478 mm/s) (Figure 2a). This locally slower flow likely contributes to the undulation of the water surface, which shows a local maximum ∼30 mm upstream of the waterfall and a local minimum at ∼80 mm (Figure 2b, Table S3 in Supporting Information S1).

We speculate that the undulating water surface profile results from a pressure drop at the waterfall lip. Where streamlines are curved, like near a waterfall lip, water pressure is not hydrostatic and instead decreases toward the center of streamline curvature (i.e., from the water surface to the bed), resulting in a gradient from higher pressure above the waterfall to lower pressure at the lip (e.g., Ota et al., 2022). The pressure gradient between the waterfall lip and a position upstream of the waterfall will be largest near the bed, allowing near‐bed flow to experience stronger acceleration than at the water surface. Thus, the pressure drop at the lip causes a deviation from a standard logarithmic vertical velocity profile, allowing a velocity reversal as observed here (Figure 2a). Particles traversing this region are likely to be influenced by the spatially variable flow field, potentially affecting particle impact rates, velocities, and, in turn, bedrock erosion.

**Figure 3:**
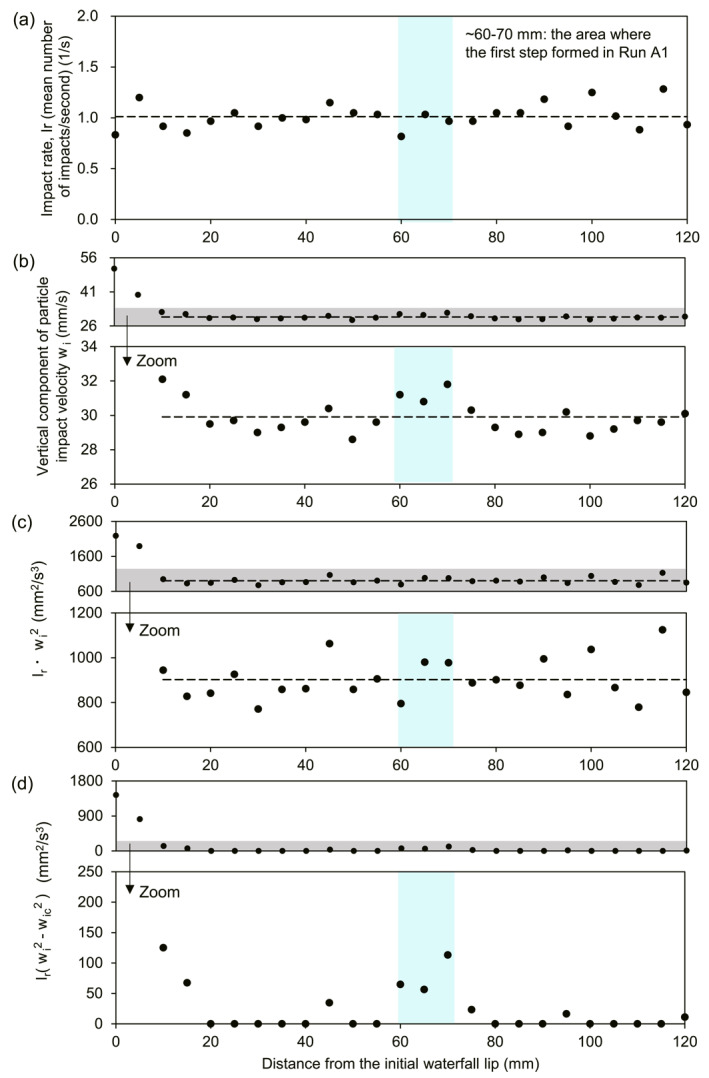
Run B1 particle impacts. (a) Mean impact rate ($I_{r}$) averaged over 1,518 impacts, (b) mean vertical particle‐impact velocity ($w_{i}$), (c) the product $I_{r}w_{i}^{2}$ and (d) the quantity $I_{r}{(w}_{i}^{2}-w_{ic}^{2})$ where $w_{ic}$ is the estimated threshold impact velocity for bedrock erosion. Dashed lines in (a), (b) and (c) are spatial means measured in (a) from 0 to 120 mm (1.01 impacts per second), and in (b) and (c) measured from 10 to 120 mm (mean impact velocity of 29.9 mm/s and mean *I*_*r*_ *w*_*i*_^*2*^ of 902.6 mm^2^/s^3^ in b and c, respectively). Panels (b), (c), and (d) show all data (top plots) and zoom in (bottom plots).

In Run B1, impact rate ranged from ∼0.8 to 1.3 impacts per second, averaging ∼1.0 impacts per second (Figure 3a, Table S4 in Supporting Information S1). The impact rate was approximately constant over the entire 120 mm section analyzed, showing no systematic increases at the position where the first step formed in Run A1 (∼60–70 mm above the waterfall). This suggests that impact rate may not strongly influence step formation.

In contrast, vertical particle impact velocity, defined as the velocity of particles moving downward 1 mm above the channel bed, was largest just upstream of the waterfall lip and was at a local maximum of ∼31.3 mm/s in the location where first step was formed in Run 1 (∼60–70 mm, Figure 3b), approximately 5% larger than the mean impact velocity between 10 and 120 mm (29.9 mm/s). The spatial distribution of impact velocity roughly matches the spatial distribution of the vertical component of flow velocity at 1 mm above the channel bed, which also locally peaks at ∼60–70 mm above the waterfall (Figures 2c and 3b).

To further explore how spatial changes in impact velocity and rate may influence bedrock erosion, we examined spatial variability in the products $\left(I_{r}w_{i}^{2}\right)$ (Figure 3c) and $\left[I_{r}\left(w_{i}^{2}-w_{ic}^{2}\right)\right]$ (Figure 3d, Table S4 in Supporting Information S1, Section 2.3). We observed high $I_{r}w_{i}^{2}$ values from 0 to 10 mm upstream of the waterfall, and approximately constant $I_{r}w_{i}^{2}$ values > 10 mm upstream of the waterfall (Figure 3c). In contrast, we observe a fundamentally different pattern when including a threshold impact velocity for bedrock erosion, that is, $\left[I_{r}\left(w_{i}^{2}-w_{ic}^{2}\right)\right]$ (Figure 3d). In this case, we set the threshold impact velocity, $w_{ic}$, to the average particle impact velocity >10 mm upstream of the waterfall ($w_{i}$ = 29.9 mm/s) based on observations of negligible erosion in this area from 0 to 60 min in Run A1 (Figure 1b). Values of $\left[I_{r}\left(w_{i}^{2}-w_{ic}^{2}\right)\right]$ peak at 0–10 mm, consistent with our Run A1 observation of rapid erosion from 0 to 10 mm upstream of the waterfall during the first 360 min. However, $\left[I_{r}\left(w_{i}^{2}-w_{ic}^{2}\right)\right]$ also locally peaks at 60–70 mm, highlighting potential for enhanced erosion in the location where the first step formed in Run A1. Together, these observations suggest that spatial changes in flow hydraulics, driven by the pressure drop at the waterfall lip, may set spatial‐variability in impact velocity and thus the location of cyclic step formation.

## 4 Influence of Changing Flow Depth

**Figure 4:**
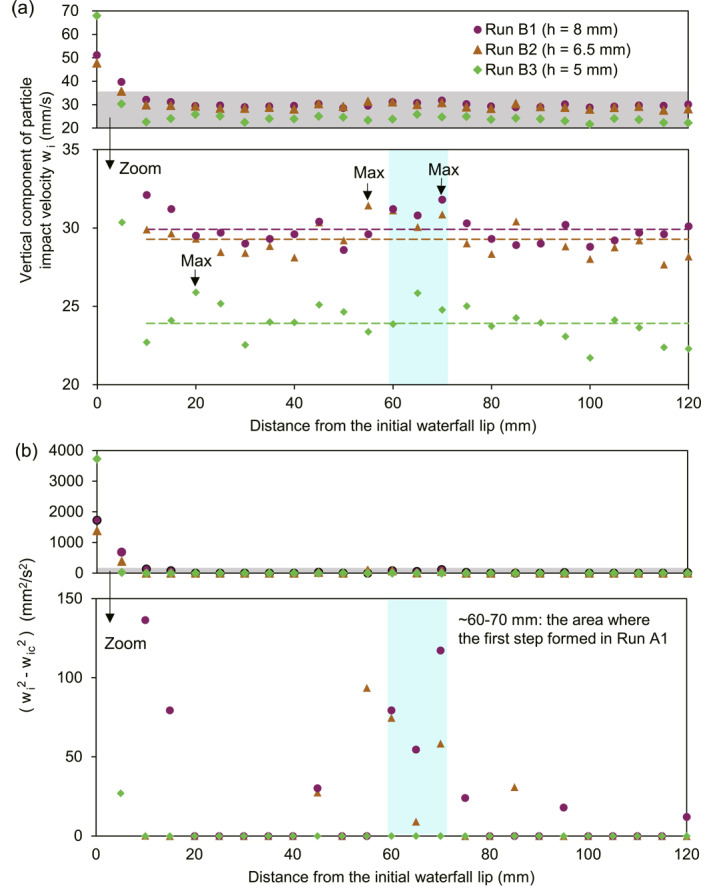
Particle impacts in Runs B1–B3. (a) Spatial distribution of the vertical particle impact velocity, *w*_*i*_, with arrows denoting the spatial location of maximum impact velocity for each Run. Arrows denote the maximum impact velocity between 20 and 120 mm upstream of the waterfall. (b) The effective impact velocity ${(w}_{i}^{2}-w_{ic}^{2})$. In both panels top plots show all data, bottom plots show zoom in. Dashed lines in bottom plot of panel (a) are the mean impact velocity from 10 to 120 mm for Runs B1–B3.

We compare Run B1 with Runs B2 and B3 to explore how reducing flow depth affects particle impacts and bedrock erosion. For Runs B2 and B3, we focus on impact velocity, rather than impact rate, because impact rate is not spatially variable in Run B1 (Figure 3a) (suggesting that step formation is driven by spatial‐variability in impact velocity, not impact rate), and furthermore because theory (Equations (1), (2), (3)) suggests bedrock erosion scales non‐linearly with impact velocity and only linearly with impact rate. We observe that decreasing flow depth decreases the spatial variability and magnitude of particle impact velocity, as well as the product [*w*_*i*_^*2*^−*w*_*ic*_^*2*^] (Figure 4, Table S4 in Supporting Information S1). In contrast to the local maxima of impact velocity and [*w*_*i*_^*2*^−*w*_*ic*_^*2*^] at ∼60–70 mm in Run B1 (Figure 4b), Run B3 shows no clear local maxima in impact velocity or [*w*_*i*_^*2*^−*w*_*ic*_^*2*^] except at the waterfall lip (Figure 4b), while Run B2 shows intermediate behavior (Figure 4b). This implies that low flow depth, as in Run B3, should preferentially promote direct waterfall‐face erosion relative to cyclic‐step development, and is consistent with the idea that decreasing flow depth causes step spacing to decrease (and eventual elimination of cyclic steps) upstream of waterfalls. In Run B3, the reduced spatial variability in [*w*_*i*_^*2*^−*w*_*ic*_^*2*^] is partly due to particle impact velocities approaching the threshold impact velocity for erosion. While *w*_*ic*_ is likely a function of bedrock strength, we note that the point of local maximum impact velocity moves toward the waterfall with decreasing flow depth, from local maxima at ∼70–∼55 mm to ∼20 mm in Runs B1, B2 and B3, respectively (Figure 4). This suggests a link between flow depth and step spacing which should promote a transition from cyclic‐step‐driven waterfall retreat to direct waterfall‐face erosion with decreasing flow depth.

Unfortunately, comparing our observations of spacing between the waterfall lip and points of locally high particle impact velocities with cyclic step theory is difficult. Existing theory (Izumi et al., 2017) and bedrock erosion models (e.g., Inoue et al., 2017, 2021; Li et al., 2020; Sumner et al., 2019, 2022) are based on the shallow‐water equation, which does not account for changes in the vertical flow velocity near the waterfall and acceleration of the flow due to pressure drop, both of which we postulate drive the spatially variable particle impact velocity in our experiments. In waterfall retreat driven by step formation, the step spacing interval can determine the waterfall retreat rate. Future experiments investigating how variations in the waterfall face angle, the upstream bed slope, and gran size (or grain size to flow depth ratio) influence step formation and spacing will be useful to build theory to accurately predict step spacing above waterfalls.

## 5 Implications

Our results suggest that decreasing flow depth tends to reduce spatial variability of erosion upstream of waterfalls and move local erosion hotspots closer to waterfall lips. We posit that this may cause a transition from cyclic‐step‐driven waterfall retreat at high discharges to retreat driven via waterfall‐face erosion at low discharges. This should occur due to two mechanisms. First, lower flow depth results in smaller particle impact velocities. If impact velocities are close to a threshold for bedrock erosion, erosion will be dominated by high‐velocity impacts at the lip where flow velocity is maximized. Second, spatial variability in flow hydraulics upstream of the waterfall lip is sensitive to flow depth. For example, Carling and Shvidchenko (2002) and Inoue et al. (2020) showed that the water surface wavelength caused by 3D antidunes increases with increasing water depth. 3D antidunes are bedforms that form in alluvial beds under supercritical flow conditions, and have some similarities to bedrock cyclic steps (e.g., the bed and water surface are in phase, often with breaking waves). This indicates that the interval of water surface fluctuations induced by the initial waterfall drop may also depend on water depth, and can help explain our observation of localized areas of high particle impact velocity moving closer to the waterfall lip with decreasing flow depth. These results suggest that, even in the absence of a threshold impact velocity for bedrock erosion, decreasing flow depth should shorten cyclic step wavelength, causing new steps to form continually closer to and eventually at the waterfall lip, in which case waterfall retreat will be driven by erosion of the waterfall face. Thus, changes in river flow depth (e.g., via climate change or anthropogenically via management practices) may result in fundamental changes in the mechanism of waterfall retreat, which can change waterfall retreat rate.

## Supporting information

Supporting Information S1

## Data Availability

All experimental data and metadata is provided in Tables S1‐S4 in Supporting Information S1 and repository (Inoue, 2024).
