# Supplementary material for: Controls on Erosion and Cyclic Step‐Formation Upstream of Waterfalls
Source: Geophys Res Lett. 2024 Nov 22;51(22):e2024GL110751. doi: 10.1029/2024GL110751 (PMC11583113; doi:10.1029/2024GL110751)
Supplement: Supplementary file 1 — Supporting Information S1 [file GRL-51-0-s001.docx]

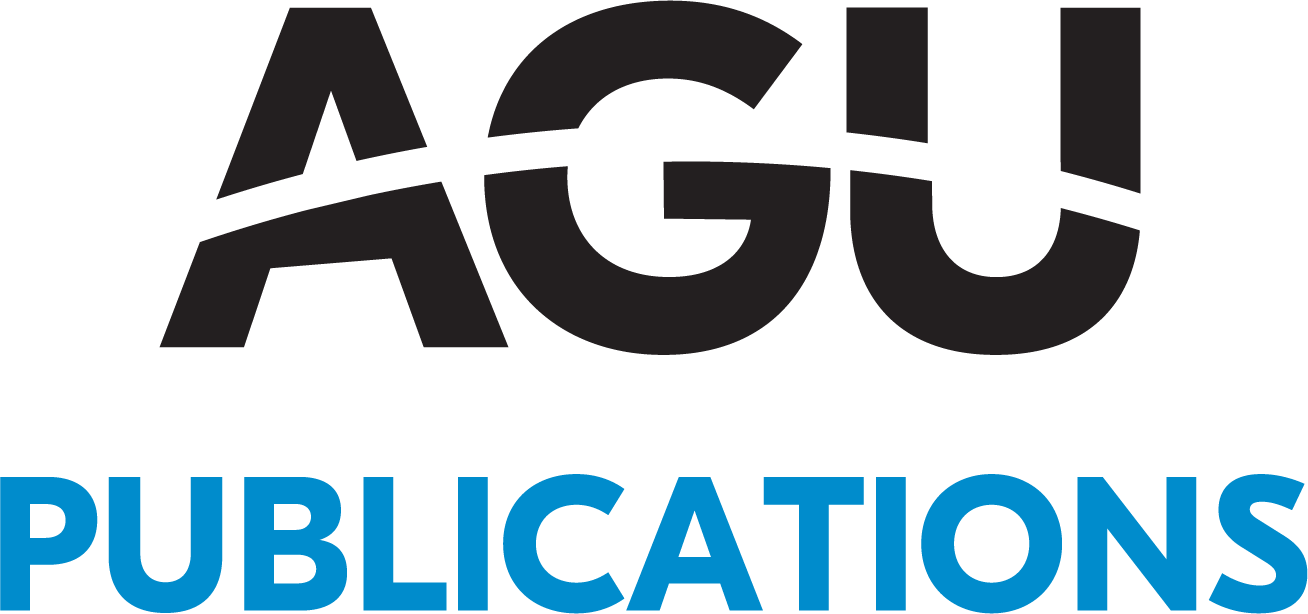


*Geophysical Research Letters*

Supporting Information for

**Controls on erosion and cyclic step-formation upstream of waterfalls**

T. Inoue^1^, Y. Hiramatsu^2^, J.S. Scheingross^3^, S. Yamaguchi^4^, K, Takahashi^1^

1. Graduate School of Advanced Science and Engineering, Hiroshima University, Hiroshima, Japan
2. Civil engineering research institute for cold region, Hokkaido, Japan
3. Department of Geological Sciences and Engineering, Nevada Geosciences, University of Nevada, Reno, USA
4. Faculty of Engineering, Hokkaido University, Hokkaido, Japan

**Contents of this file**

Text S1, S2 and Table S1

**Additional Supporting Information (Files uploaded separately)**

Table S2 to S4

**Introduction**

This Supporting Information file contains methods on Particle Tracking Velocimetry (Text S1), Particle Image Velocimetry (Text S2), measurement of water surface slope (Text S2), and a table providing an overview of the different experiments (Table S1).

Text S1: Measurement of particle impact velocity and frequency using Particle Tracking Velocimetry (PTV)

For Runs B1 – B3 we used a Fastcam SA3 high-speed camera with a SIGMA 24 mm lens to film saltating sand particles (grain diameter, $D$ = 1.4 mm) introduced at the upstream end of the flume. We held the distance from the tip of the camera to the side of the channel constant at 200 mm for all filming with a constant frame rate of 500 frames per second, 1/1000 s shutter speed, and aperture value of 4. We tilted the camera at an angle equal to the riverbed slope so that the channel bed appeared horizontal in the camera image and filmed in 60 s durations. A reflector lamp installed above the channel provided a light source.

We used a commercial system (Flow Expert by KATO KOKEN) for PTV analysis. After calculating the velocity vector of each particle using binary correlation analysis, we calculated the time-averaged velocity of particles passing through a 1 mm x 1 mm grid set that extended from the channel bed to the water surface. In calculating the time-averaged particle velocity, we only counted particles with a downward component of vertical motion (i.e., particles impacting the channel bed). We measured the particle impact velocity in Runs B1, B2, and B3, and we additionally measured the frequency of particle impacts in Run B1 only.

Measuring the particle impact frequency was a laborious task which we performed by manually tracking 265 particles frame-by-frame through the video images taken for PTV analysis. We focused on the section of the flume from the waterfall lip to a point 120 mm upstream of the lip and we manually recorded the location and time that each of the 265 particles impacted the bed, allowing us to calculate the frequency of particle impacts as a function of distance upstream of the waterfall lip.

Text S2: Measurement of the flow field and water surface profile using Particle Image Velocimetry (PIV)

We measured the flow field for Run B1 only using 0.49 mm diameter resin particles (Mitsubishi Chemical Group: DIAION HP20) with a specific gravity of 1.01 which we sprayed in from the upstream end of the flume at a time at a time with zero siliciclastic sediment transport. We used a laser-sheet light source (DPGL-8W by Japan Laser) irradiating vertically downward from above the channel, and, in an attempt to capture the flow in a vertical 2-dimensional plane, we photographed particles from the channel sidewall. We used the direct cross-correlation method in Flow Expert by KATO KOKEN for PIV analysis with a 26-pixel (~4.99 mm) inspection area size, a 20-pixel (~3.84 mm) vertical and horizontal exploration area, and other settings left at their default values. For Run B1 only, we additionally measured water surface slope for 20 randomly selected PIV analysis images by taking advantage of the contrast between the high luminance of the water surface reflecting the laser sheet was high and the low luminance above the water surface. Note that PIV and PTV may not give identical measurements. Unlike PTV which tracks individual particles, PIV tracks the behavior of groups of particles. For our PTV analysis, we analyze only downward moving grains; whereas PIV analysis includes both descending and ascending flow. This may result in lower flow velocities (measured with PIV) relative to impact velocities (measured with PTV on only the downward moving grains).

**Table S1:** Summary of experimental parameters

|  | Run A1 | Run B1 | Run B2 | Run B3 |
| --- | --- | --- | --- | --- |
| Channel width (cm) | 1 | 1 | 1 | 1 |
| Channel slope | 0.02 | 0.02 | 0.02 | 0.02 |
| Waterfall drop height (cm) | 5 | 5 | 5 | 5 |
| Sediment size (mm) | 1.4 | 1.4 | 1.4 | 1.4 |
| Sediment supply rate (ml/min) | 6.7 | 6.7 | 6.7 | 6.7 |
| Bed roughness height (mm) | 0.54 | 0.54 | 0.54 | 0.54 |
| Flow discharge (l/s) | 0.038 | 0.038 | 0.027 | 0.0175 |
| Normal flow depth (mm) | 8 | 8 | 6.5 | 5 |
| Normal flow velocity (cm/s) | 47.5 | 47.5 | 41.5 | 35 |
| Froude number | 1.7 | 1.7 | 1.64 | 1.57 |
| Reynolds number | 3800 | 3800 | 2700 | 1750 |
| Reynolds particle number | 55 | 55 | 50 | 44 |
| Transport stage (τ_*_⁄τ_*c_)* | 4.1 | 4.1 | 3.3 | 2.6 |
| Sediment supply-capacity ratio (*q_s_/q_sc_*) | 0.33 | 0.33 | 0.51 | 0.93 |
| Bed condition | Erodible | Non-erodible | Non-erodible | Non-erodible |

*In calculating τ_*_, the roughness of the sidewalls is ignored for simplicity.

**Tables S2 to S4**

Tables S2 to S4 contain all experimental data and are uploaded as a separate excel file for ease of access. (DOI: 10.5281/zenodo.11646982)

**Table S2:** Longitudinal profiles for Run A1

**Table S3:** Water surface profile for Run B1

**Table S4:** Tabulated data of impact rate, impact velocity, and flow velocity as a function of distance from the waterfall for Runs B1 - B3
